# Correlation Between Accommodative Facility and Light-Evoked Pupil Responses in Individuals with History of Mild Traumatic Brain Injury

**DOI:** 10.3390/jemr19030065

**Published:** 2026-06-08

**Authors:** Curt Fritts-Davis, Andrew T. E. Hartwick, Marjean T. Kulp

**Affiliations:** College of Optometry, Ohio State University, 338 W 10th Ave, Columbus, OH 43210, USA; curtfrittsdavis@gmail.com (C.F.-D.); hartwick.4@osu.edu (A.T.E.H.)

**Keywords:** brain concussion, pupil, accommodation, ocular, brain injuries, traumatic, vision disorders

## Abstract

Introduction: Previous studies have shown those with a history of a traumatic brain injury (TBI) have altered pupillary light responses compared with those without a history of TBI. Those with a history of TBI are also more likely to have accommodative deficits. We investigated the relationship between light-evoked pupil dynamics and accommodative function in individuals who have previously experienced a TBI. Methods: A total of 17 participants with a history of mild TBI were recruited. Pupil metrics were measured using a commercial pupillometer and included baseline diameter, latency, constriction amplitude, average constriction velocity, peak constriction velocity and peak dilation velocity. Accommodative function was assessed using clinical measurements of facility and amplitude. Pupil metrics were compared among those with versus without accommodative dysfunction. Results: One-way ANCOVA testing (controlling for age and time since most recent TBI) comparing groups with and without accommodative dysfunction showed that those with accommodative dysfunction had significantly larger light-evoked pupil constriction amplitudes (*p* = 0.037) and significantly faster average constriction velocity (*p* = 0.007) compared with those without accommodative dysfunction. No significant differences were observed for other pupil metrics (*p* > 0.05 for all). ANCOVA testing (controlling for age and time since TBI) to determine whether decreased amplitude of accommodation or facility was more strongly related to the differences in pupil metrics observed between those with versus without accommodative dysfunction, showed significantly larger light-evoked pupil constriction amplitudes (*p* = 0.007) and significantly faster average constriction velocity (*p* = 0.002) among those with reduced accommodative facility compared with those with normal accommodative facility. No statistically significant differences were observed between those with reduced versus normal accommodative amplitude (*p* ≥ 0.07). Among all participants, monocular accommodative facility measures were significantly correlated with greater pupil constriction amplitude (right eye: rho = −0.721, *p* = 0.001; left eye: rho = −0.65, *p* = 0.005), and greater average constriction velocity (right eye: rho = −0.58, *p* = 0.015; left eye: rho = −0.57, *p* = 0.016). Conclusions: The results of this small-sample study suggest that accommodative function and light-evoked pupillary dynamics are correlated in individuals with a history of TBI. Those with accommodative dysfunction showed greater pupil constriction amplitudes and velocities and this relationship may reflect shared autonomic or oculomotor mechanisms.

## 1. Introduction

Traumatic brain injury (TBI) results in hospitalization for 7 out of every 10,000 American civilians every year [1]. Up to 90% of all TBIs are classified as “mild”, a designation that relates to the duration in loss of memory and consciousness at the time of an injury rather than the severity and persistence of symptoms [2,3]. In fact, the visual system is often chronically impacted for years in individuals with mild TBI [4,5], and clinical ocular testing can objectively show persistent deficits. Two of the physiological processes that have been shown to be altered after mild TBI (also known as concussion) include accommodative function (ability to focus on near targets) and pupillary light responses (change in pupil size with light stimulation). Accommodative dysfunction has been shown to be much more common in individuals with TBI (32–57%) [4,6,7,8,9,10,11] as compared with the general population (2%) [12]. Although there are some discrepancies in the literature on which pupillary response parameters are most affected after concussion, multiple studies involving adults and children with a history of TBI have reported altered pupillary dynamics (magnitude and speed of pupil constriction/dilation), relative to healthy controls [13,14,15,16,17,18,19].

Accommodation and pupil function are intrinsically linked together as part of the near response triad [20]. This triad is largely controlled by the autonomic nervous system, which has been shown to be dysfunctional after a concussion [21]. The sympathetic and parasympathetic innervation of the iris and ciliary muscle that drives both accommodation and pupil constriction/dilation shares the same efferent pathways.

Despite the shared aspects of the neural circuitry regulating accommodation and pupils, and the reported effect of mild TBI on altered pupillary dynamics and accommodative function in prior studies, the relationship between changes in accommodative and pupillary function after head injury has not been previously investigated to our knowledge. Therefore, the purpose of this small-sample study was to examine the correlation between light-evoked pupil dynamics and accommodative function in a cohort of individuals who had previously experienced a mild TBI.

## 2. Methods

This cross-sectional study was conducted at The Ohio State University College of Optometry in Columbus, Ohio. This research was reviewed by an independent ethical review board and conforms with the principles and applicable guidelines for the protection of human subjects in biomedical research. This study followed the tenets of the Declaration of Helsinki, and the institutional review board approved the protocol and informed consent forms (OSU IRB #2020H0235). Adult participants completed informed consent and Health Insurance Portability and Accountability Act (HIPAA) authorization; the parent or guardian of each participating child provided written parental permission, and each child participant provided written assent.

### 2.1. Participants

Participants with a reported history of at least one, physician-diagnosed TBI were recruited. The Ohio State University TBI Identification Method Interview Form, which has been validated in previous studies, was used to characterize the severity of the TBI [22,23]. Because prior studies have shown altered accommodative and pupillary function in individuals with prior TBI, along with a significant range of values for these measurements, only participants with a history of TBI were recruited. Participants were at least 7 years old to ensure they could understand and participate in all testing procedures and no older than 30 years old to ensure accommodative changes were not a result of incipient presbyopia. All participants wore appropriate refractive correction for at least 2 weeks prior to study participation. Participants were required to show a best corrected distance visual acuity of 0.1 logMAR (6/7.5) or better in each eye. Participants were not eligible if they had a history of eye muscle surgery, seizures when exposed to flashing lights, Graves’ thyroid disease, multiple sclerosis, myasthenia gravis, diabetes, or Parkinson disease. Participants also were excluded if they had a lifetime history of any self-reported non-traumatic acquired brain injury including stroke, brain tumor, brain aneurysm, encephalitis, meningitis, heart attack, or near drowning experience.

### 2.2. Procedures

**Figure 1:**
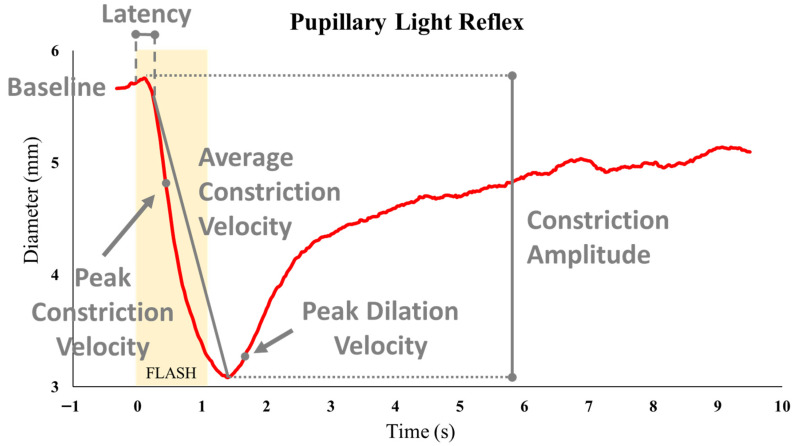
Typical trace showing pupil diameter before, during, and after a one-second pulse of white light and the measured parameters are indicated. Baseline diameter is the average pupil diameter measured during the 300 ms before stimulus onset. Latency is the time interval between stimulus onset and the first point where the diameter is less than 97% of the baseline value. Constriction amplitude is the difference between the maximum and minimum pupil diameter values. Average constriction velocity is the slope of the line connecting the last point of the latency period and the minimum diameter point. Peak constriction velocity is the steepest slope of the pupil trace between the last point of the latency period and the minimum diameter point. Peak dilation velocity is the steepest slope of the pupil trace after the minimum diameter point.

Binocular pupil function was assessed by continuously recording pupil diameter during a one-second flash of white light followed by nine seconds of darkness. A RAPDx pupillometer (Konan Medical, Irvine, CA, USA) was used in a dark windowless room to present the stimulus and record images of both pupils at the same time. The RAPDx uses an infrared camera to record pupil diameter at a rate of 40 Hz. The binocular white light stimulus used to evoke pupil constriction had an illumination of approximately 80 lux at each corneal plane. Recordings began 300 ms before the 1 second flash of light to acquire baseline pupil size before stimulus onset. Pupil diameter data was smoothed using a 10-datapoint moving average before any metrics were calculated. The following metrics were then calculated using a MATLAB program (R2021a) using the time and pupil diameter data collected by the pupillometer: baseline diameter, latency, constriction amplitude, average constriction velocity, peak constriction velocity and peak dilation velocity (see Figure 1). Symptoms were evaluated with both the Convergence Insufficiency Symptom Survey, which is frequently used to assess symptom severity during sensorimotor exams and the Brain Injury Vision Symptom Survey which has been shown to have a high sensitivity for TBI symptoms [24,25,26].

Monocular accommodative amplitude was measured using a push-up technique with a Gulden near point rule (Gulden Ophthalmics, Elkins Park, PA, USA). A single column of 0.2 logMAR (6/9.5) letters placed initially at 40 cm was moved slowly toward the participant at a speed of 1–2 cm/second until the participant reported first sustained blur. Monocular accommodative amplitude was recorded as the distance from the forehead to the point at which the participant reported first sustained blur. The distance was measured to the nearest half centimeter and then converted to diopters. This procedure was performed monocularly for the right eye, and then again for the left eye. Monocular accommodative facility was performed while the participant looked at a single column of 0.2 logMAR (6/9.5) letters on a Gulden fixation stick (Gulden Ophthalmics, Elkins Park, PA, USA) at 40 cm. The examiner held a +2.00 diopters (D) lens in front of the open eye and instructed the participant to clear the target. When the participant indicated the target was clear, the examiner switched the lens in front of the open eye to −2.00 D. After a brief demonstration, this was repeated as many times as possible in one minute (recorded as flips per minute).

### 2.3. Statistical Analyses and Classification of Accommodative Dysfunction

Participants also were divided into two groups, those with accommodative dysfunction and those without accommodative dysfunction, for statistical analysis. Accommodative dysfunction was classified as having: (1) an amplitude of accommodation in either eye that was ≥2 D less than Hofstetter’s minimum expected value (15 − ¼ × age) and/or (2) monocular accommodative facility that was ≤12 flips per minute in either eye. These criteria were based upon those used in prior studies [6,7,27] and are commonly used in clinical practice [28]. The right eye pupil metrics for those with versus without accommodative dysfunction were compared using one-way nonparametric ANCOVA controlling for age and time since TBI. Symptom scores, number of TBIs, and time since TBI for those with versus without accommodative dysfunction were compared using one-way nonparametric ANCOVA controlling for age. Pupil metrics and clinical accommodative measures were compared using Spearman’s correlation.

## 3. Results

The study enrolled 17 participants, 12 of whom were female. The average age was 22.9 (±5.6) years with a range of 10 to 29 years old. Each participant reported at least one prior TBI, with the most common causes of the most recent TBI being a motor vehicle accident or sports-related concussion (Table 1 and Table S1). Every TBI reported by the participants was confirmed to be mild in severity by using The Ohio State University TBI Identification Method Interview Form to document the prior head injuries. Based on established criteria (see Section 2), nine participants were diagnosed with accommodative dysfunction and eight participants were not. Of the nine that exhibited accommodative dysfunction, three had reduced facility, one had reduced amplitude, and five had both amplitude and facility deficits.

One-way ANCOVA statistical testing (controlling for age and time since most recent TBI) comparing groups with and without accommodative dysfunction showed that those with accommodative dysfunction had significantly larger light-evoked pupil constriction amplitudes (*p* = 0.037) and significantly faster average constriction velocity (*p* = 0.007) compared with those without accommodative dysfunction. No significant differences were observed for other pupil metrics (baseline diameter, latency, peak constriction velocity, and peak dilation velocity; *p* > 0.05 for all). Because accommodative function changes with age, we also performed an analysis limited to those aged 18 to 30 years; we again found that those with accommodative dysfunction had significantly larger light-evoked pupil constriction amplitudes (*p* = 0.019) and significantly faster average constriction velocity (*p* = 0.016) compared with those without accommodative dysfunction. Paired sample testing indicated that pupil parameters were not significantly different between the two eyes (*p* > 0.05).

**Figure 2:**
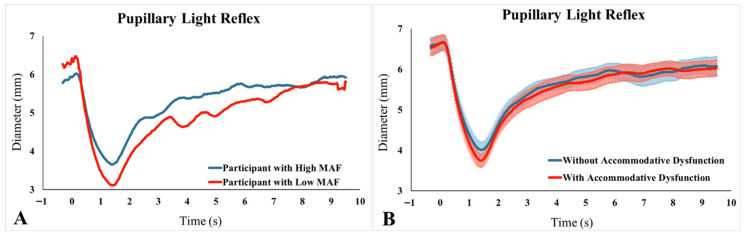
(**A**) Pupil traces from two representative participants to demonstrate differences in pupil behavior in participants with disparate accommodative function. The two participants represented here were chosen because they had the highest and lowest MAF (monocular accommodative facility) values, respectively. (**B**) Average pupil traces of those with and without accommodative dysfunction. The shaded areas represent standard error.

The average traces of pupil size in those with and without accommodative dysfunction illustrate the overall difference in pupil constriction (Figure 2B) and traces from example participants with the highest and lowest monocular accommodative facility (MAF) highlight the difference in pupillary dynamics (Figure 2A).

Symptom survey scores measured with CISS and BIVSS were higher on average for those with versus without accommodative dysfunction, but the differences were not statistically significant (*p* > 0.05) (Table 2).

ANCOVA testing (controlling for age and time since TBI) to determine whether decreased amplitude of accommodation or facility was more strongly related to the differences in pupil metrics observed between those with versus without accommodative dysfunction, showed significantly larger light-evoked pupil constriction amplitudes (*p* = 0.007) and significantly faster average constriction velocity (*p* = 0.002) among those with reduced accommodative facility compared with those with normal accommodative facility. No statistically significant differences were observed between those with reduced versus normal accommodative amplitude (*p* ≥ 0.07).

**Figure 3:**
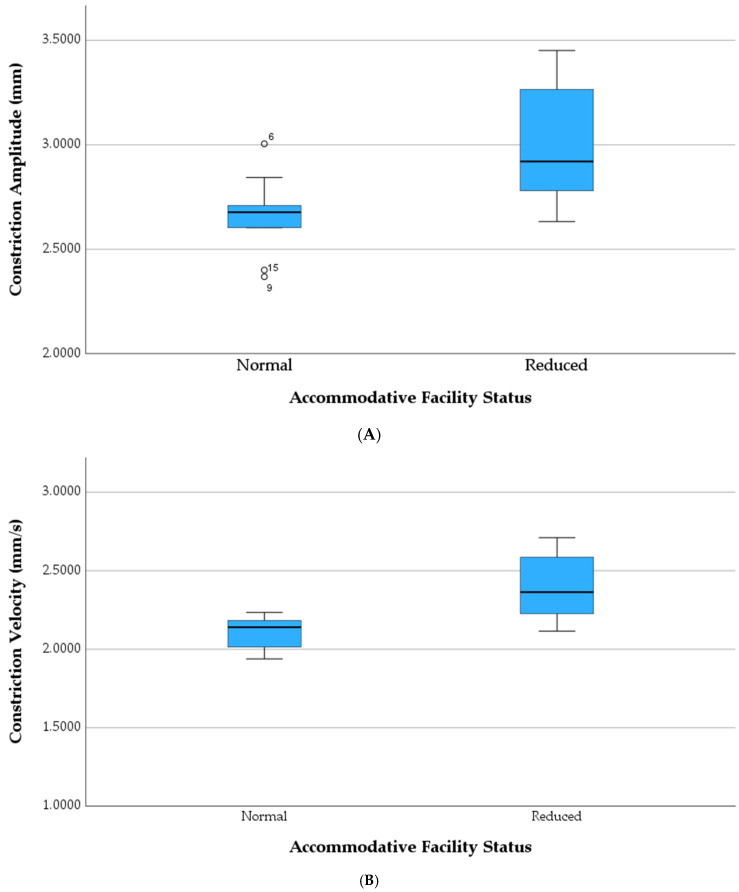
Box plots summarizing group distributions of pupil measures for those with versus without reduced accommodative facility (flips per minute). (**A**) Constriction amplitude (mm) (outlier labels are participant numbers, not test values); (**B**) constriction velocity (mm/s).

**Figure 4:**
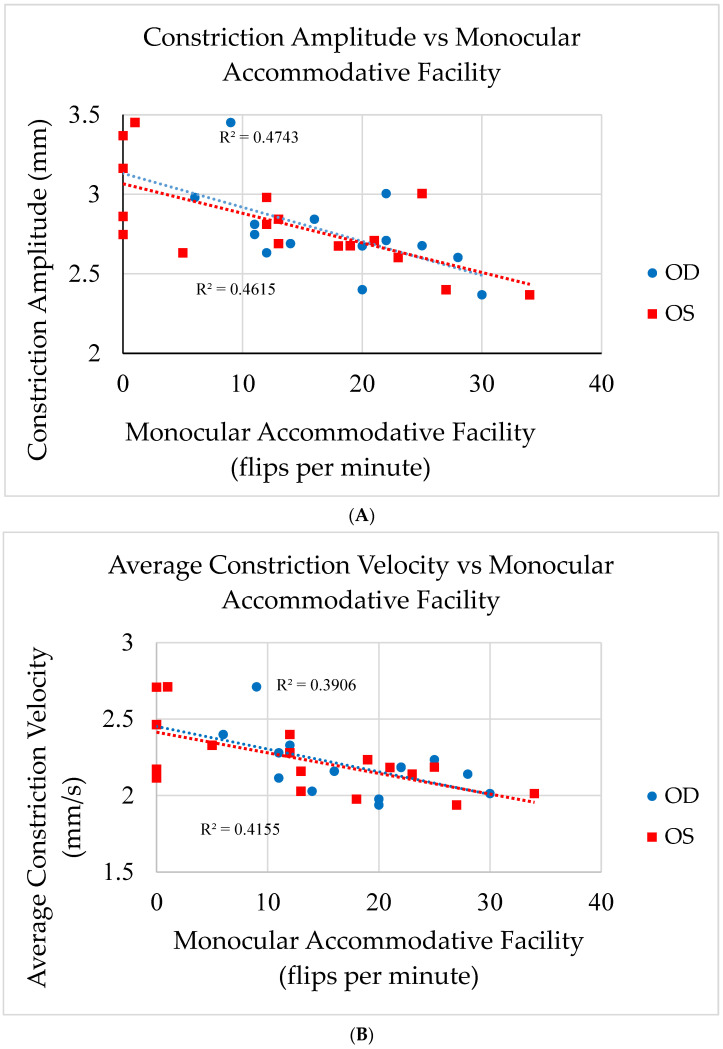
Scatter plots to illustrate correlation between pupil metrics and accommodative facility. (**A**) Constriction amplitude (mm); (**B**) constriction velocity (mm/s).

Figure 3 shows box plots summarizing group distributions of pupil constriction amplitude and velocity for those with versus without reduced accommodative facility. Scatter plots display relationships between accommodative facility and pupil measures (Figure 4). Among all participants, monocular accommodative facility measures were significantly correlated with greater pupil constriction amplitude (right eye: rho = −0.721, *p* = 0.001; left eye: rho = −0.65, *p* = 0.005), and greater average constriction velocity (right eye: rho = −0.58, *p* = 0.015; left eye: rho = −0.57, *p* = 0.016).

## 4. Discussion

While several studies have reported altered pupillary response dynamics and altered accommodative function in individuals with prior head injury [29,30], these studies have not typically assessed both parameters. As one exception, Dutta and colleagues [29] measured aspects of pupillary and accommodative function in a group of 63 individuals with history of mild TBI and found that both ocular functions were significantly altered in this group, relative to a group of age-matched controls. While this prior study provided evidence that both pupillary and accommodative dysfunction can be present in individuals with prior head injury, it did not report on whether there was a relationship between pupillary and accommodative function in their study group [13]. As prior studies have already established alterations in accommodation and pupillary responses in individuals with TBI relative to control comparison groups, the current study focused on investigating the correlation of parameters related to these ocular functions in a sample exclusively of participants with TBI history. Individuals with prior TBI have been shown to have greater variability in the amplitude of pupil constriction evoked by single [18] and repeated light pulses [31]. Similarly, a comparison group involving individuals without a binocular vision disorder would be expected to have a narrower range of accommodative amplitude and dysfunction values [27,28], thus limiting the likelihood of finding a correlation.

All of the participants in our cohort had a history of mild TBI, and 53% of them met the diagnostic criteria for accommodative dysfunction. This finding of a relatively high prevalence of accommodative disorders in individuals after head injury is consistent with the prior literature [4,6,7,8,9,10,11]. We found that those classified as having accommodative dysfunction showed significantly larger pupil constriction amplitudes and faster average constriction velocities than those without accommodative dysfunction. This relationship was maintained when comparing those with versus without reduced accommodative facility, but differences were not statistically significant for reduced accommodative amplitude. Additionally, accommodative facility was significantly correlated with larger pupil constriction amplitudes and faster average constriction velocities.

Our finding that increased pupil reactivity (amplitude and velocity of constriction) correlated to reduced accommodative facility was somewhat unexpected. While it has been reported that reduced light-evoked pupil amplitudes and constriction velocities occur in individuals with TBI relative to controls [32], our findings do not contradict those results because accommodative function was not assessed in those studies. Specifically, we found that within a cohort of individuals with prior mild TBI, the individuals without accommodative dysfunction had smaller light-evoked pupil amplitudes and constriction velocities compared with those with accommodative dysfunction.

The exact mechanism that underlies this relationship remains unknown, but it is possible that alterations in the sympathetic/parasympathetic tone that regulates pupil size and accommodation can occur after head injury [21]. Manipulation of sympathetic and parasympathetic innervation of the pupil in rhesus monkeys changed its light-evoked constriction amplitude and velocity. Specifically, the increase in the influence of parasympathetic innervation by destroying sympathetic innervation pathways resulted in larger constriction amplitude and faster constriction velocity, while decreasing parasympathetic innervation had the opposite effect [33]. In a previous study involving a large (>250) cohort of children and adolescents who were classified as having either increased sympathetic tone, increased parasympathetic tone or neutral tone based on cardiac measures (Kerdo index), the minimum pupil size after light stimulation was significantly smaller in the group with increased parasympathetic tone [34]. A more recent study performed on elite athletes and controls also sheds light on the parasympathetic nervous system’s influence on pupil behavior, as the athletes exhibiting higher parasympathetic tone had larger light-evoked pupil constriction amplitudes and faster constriction velocities both at rest and at maximum exertion, relative to a comparison control group [35]. Taken together, these studies support the hypothesis that the individuals with prior TBI also may have had increased parasympathetic tone, due to the presence of larger and faster light-evoked pupil constriction. However, it should be noted that there were no observed differences in baseline pupil size between the groups with versus without accommodative dysfunction in this study. Instead of basal differences in autonomic tone, it is possible that the iris muscle is more sensitive to the parasympathetic stimulation it receives from the efferent arm of the pupillary light reflex in individuals with TBI and accommodative dysfunction. As both the ciliary muscle (controls accommodation) and the iris sphincter (controls pupil constriction) receive parasympathetic innervation from short ciliary nerves that project from the ciliary ganglion [36], it is unclear how an inverse relationship between accommodation and pupil constriction (reduced facility of the former correlated to increased velocity and amplitude with the latter) could occur if the function of the efferent parasympathetic neural circuit to the eye was altered after a head injury. It should be noted that this study cannot determine whether the observed association is specific to TBI or whether a similar association may also exist in individuals with accommodative dysfunction without a TBI history. Future studies are planned to investigate this potential mechanism further, including studies on pupillary function on individuals diagnosed with accommodative dysfunction but not having a TBI history.

A limitation of this study is the relatively small sample size, and the correlation noted here should be assessed in larger cohorts and in individuals during both pre-TBI and post-TBI testing. While the push-up method is commonly used clinically, it has been shown to overestimate accommodative amplitude due to depth of focus and changes in angular target size [37]. Additionally, the results from this study are not generalizable to populations with moderate or severe TBI; however, approximately 90% of all TBIs are classified as mild [2,3]. Finally, larger constriction amplitude and faster average constriction velocity may be related and may not represent independent autonomic markers.

In summary, our results indicate that accommodative dysfunction and light-evoked pupillary dynamics are significantly related in individuals with a prior history of mild TBI. The mechanism linking enhanced pupil constriction in individuals with TBI and accommodative dysfunction remains unknown, however it may involve changes to autonomic tone and enhanced parasympathetic input in these individuals. In future work, it would be interesting to record pupil metrics before, during, and after vision therapy treatment for accommodative dysfunction following TBI and assess whether this correlation persists in individuals exhibiting improvement in accommodative facility. With respect to sports-related concussion, future studies could also compare pupil and accommodative parameters measured during preseason testing versus testing following impact exposure, as the obtained objective data may aid in return-to-play decisions.

## Figures and Tables

**Table 1:** TBI characteristics and accommodative status for study participants.

		Causes of TBI							
ID	Age (Years)	Motor Vehicle Accident (MVA)	Fall	Sports- Related Concussion (SRC)	Struck by Object (SBO)	Total TBIs	Most Recent (Years Ago)	Most Recent (Cause)	Accommodative Dysfunction
1	10	1	1			2	1.25	MVA	Yes
2	11		1	1	1	3	3.00	Fall	No
3	25	1		3		4	1.00	MVA	No
4	28	1	1			2	2.00	MVA	No
5	28	1	2		2	5	1.33	MVA	Yes
6	18				2	2	1.00	SBO	Yes
7	26			3		3	2.00	SRC	No
8	25	1				1	1.00	MVA	Yes
9	24		1			1	5.00	Fall	No
10	23			1	3	4	1.00	SBO	No
11	29	2		5	1	8	3.00	SBO	Yes
12	15	1	1	2		4	0.25	SRC	Yes
13	22			1		1	5.00	SRC	No
14	23			1		1	7.00	SRC	Yes
15	19			1	1	2	1.00	SRC	Yes
16	23	1	1	2		4	5.00	MVA	No
17	23	1				1	3.00	MVA	Yes
	Total:	10	8	20	10	48			

**Table 2:** Associations between accommodative status and symptoms and prior TBI.

Accommodative Dysfunction	N	%	Age	CISS	BIVSS	Number of TBIs	Years Since Last TBI
With Diagnosis	9	52.9	21.1 ± 2.1	23 ± 2	29 ± 4	3.1 ± 0.8	2.1 ± 0.7
Without Diagnosis	8	47.1	22.8 ± 1.8	17 ± 2	23 ± 4	2.6 ± 0.5	3.0 ± 0.6
ANCOVA (controlling for age) (*p*-value)				0.19	0.085	0.78	0.287

## Data Availability

The original contributions presented in this study are included in the article and Supplementary Materials. Further inquiries can be directed to the corresponding author upon reasonable request.

## Supplementary Materials

The following supporting information can be downloaded at: https://www.mdpi.com/article/10.3390/jemr19030065/s1, Table S1: Accommodative Facility and Light-Evoked Pupil Responses.

## References

[1] Peterson A.B. (2021). Incidence of Nonfatal Traumatic Brain Injury–Related Hospitalizations—United States, 2018. MMWR Morb. Mortal. Wkly. Rep..

[2] Leo P., McCrea M., Laskowitz D., Grant G. (2016). Epidemiology. Translational Research in Traumatic Brain Injury.

[3] Holm L., Cassidy J.D., Carroll L.J., Borg J. (2005). Neurotrauma Task Force on Mild Traumatic Brain Injury of the WHO Collaborating Centre Summary of the WHO Collaborating Centre for Neurotrauma Task Force on Mild Traumatic Brain Injury. J. Rehabil. Med..

[4] Capó-Aponte J.E., Jorgensen-Wagers K.L., Sosa J.A., Walsh D.V., Goodrich G.L., Temme L.A., Riggs D.W. (2017). Visual Dysfunctions at Different Stages after Blast and Non-blast Mild Traumatic Brain Injury. Optom. Vis. Sci..

[5] Barnett B.P., Singman E.L. (2015). Vision Concerns After Mild Traumatic Brain Injury. Curr. Treat. Options Neurol..

[6] Master C.L., Scheiman M., Gallaway M., Goodman A., Robinson R.L., Master S.R., Grady M.F. (2016). Vision Diagnoses Are Common After Concussion in Adolescents. Clin. Pediatr..

[7] Scheiman M., Grady M.F., Jenewein E., Shoge R., Podolak O.E., Howell D.H., Master C.L. (2021). Frequency of oculomotor disorders in adolescents 11 to 17 years of age with concussion, 4 to 12 weeks post injury. Vis. Res..

[8] Gallaway M., Scheiman M., Mitchell G.L. (2017). Vision Therapy for Post-Concussion Vision Disorders. Optom. Vis. Sci..

[9] Brahm K.D., Wilgenburg H.M., Kirby J., Ingalla S., Chang C.-Y., Goodrich G.L. (2009). Visual Impairment and Dysfunction in Combat-Injured Servicemembers With Traumatic Brain Injury. Optom. Vis. Sci..

[10] Peiffer A.J., MacDonald J., Duerson D., Mitchell G., Hartwick A.T.E., McDaniel C.E. (2020). The Influence of Binocular Vision Symptoms on Computerized Neurocognitive Testing of Adolescents With Concussion. Clin. Pediatr..

[11] Marusic S., Vyas N., Chinn R.N., O’Brien M.J., Roberts T.L., Raghuram A. (2024). Vergence and accommodation deficits in paediatric and adolescent patients during sub-acute and chronic phases of concussion recovery. Ophthalmic Physiol. Opt..

[12] Scheiman M., Gallaway M., Coulter R., Reinstein F., Ciner E., Herzberg C., Parisi M. (1996). Prevalence of vision and ocular disease conditions in a clinical pediatric population. J. Am. Optom. Assoc..

[13] Dutta P., Vittal S., Selvakumar A., Atiya A., Hussaindeen J.R. (2023). Pupillary dynamics and accommodative response in mild traumatic brain injury. Taiwan J. Ophthalmol..

[14] Carrick F.R., Azzolino S.F., Hunfalvay M., Pagnacco G., Oggero E., D’Arcy R.C.N., Abdulrahman M., Sugaya K. (2021). The Pupillary Light Reflex as a Biomarker of Concussion. Life.

[15] Hsu J., Stec M., Ranaivo H.R., Srdanovic N., Kurup S.P. (2021). Concussion Alters Dynamic Pupillary Light Responses in Children. J. Child Neurol..

[16] Master C.L., Podolak O.E., Ciuffreda K.J., Metzger K.B., Joshi N.R., McDonald C.C., Margulies S.S., Grady M.F., Arbogast K.B. (2020). Utility of Pupillary Light Reflex Metrics as a Physiologic Biomarker for Adolescent Sport-Related Concussion. JAMA Ophthalmol..

[17] Truong J.Q., Ciuffreda K.J. (2016). Comparison of pupillary dynamics to light in the mild traumatic brain injury (mTBI) and normal populations. Brain Inj..

[18] Thiagarajan P., Ciuffreda K.J. (2015). Pupillary responses to light in chronic non-blast-induced mTBI. Brain Inj..

[19] Capó-Aponte J., Urosevich T., Walsh D., Temme L., Tarbet A. (2013). Pupillary Light Reflex as an Objective Biomarker for Early Identification of Blast-Induced mTBI. Spine.

[20] Rucker J.C., Buettner-Ennever J.A., Straumann D., Cohen B. (2019). Case Studies in Neuroscience: Instability of the visual near triad in traumatic brain injury—Evidence for a putative convergence integrator. J. Neurophysiol..

[21] Mercier L.J., Batycky J., Campbell C., Schneider K., Smirl J., Debert C.T. (2022). Autonomic dysfunction in adults following mild traumatic brain injury: A systematic review. NeuroRehabilitation.

[22] Bogner J., Corrigan J.D. (2009). Reliability and predictive validity of the Ohio State University TBI identification method with prisoners. J. Head Trauma Rehabil..

[23] Corrigan J.D., Bogner J. (2007). Initial reliability and validity of the Ohio State University TBI Identification Method. J. Head Trauma Rehabil..

[24] Laukkaned H., Scheiman M., Hayes J.R. (2017). Brain Injury Vision Symptom Survey (BIVSS) Questionnaire. Optom. Vis. Sci..

[25] Rouse M., Borsting E., Mitchell G.L., Cotter S.A., Kulp M., Scheiman M., Barnhardt C., Bade A., Yamada T., Yamada T. (2009). Validity of the convergence insufficiency symptom survey: A confirmatory study. Optom. Vis. Sci..

[26] Rouse M.W., Borsting E.J., Mitchell G.L., Scheiman M., Cotter S.A., Cooper J., Kulp M.T., London R., Wensveen J. (2004). Validity and reliability of the revised convergence insufficiency symptom survey in adults. Ophthalmic Physiol. Opt..

[27] Kulp M.T., Sinnott L.T., Cotter S.A., Borsting E., Toole A.J., Chen A.M., Jenewein E.C., Morrison A.M., Plaumann M.D., Jones-Jordan L. (2022). Does coexisting accommodative dysfunction impact clinical convergence measures, symptoms and treatment success for symptomatic convergence insufficiency in children?. Ophthalmic Physiol. Opt..

[28] Scheiman M., Wick B. (2019). Clinical Management of Binocular Vision.

[29] Dutta P., Baishya R. (2024). Pupillary dynamics, accommodation and vergence in concussion. Clin. Exp. Optom..

[30] Thiagarajan P., Ciuffreda K.J. (2022). Accommodative and pupillary dysfunctions in concussion/mild traumatic brain injury: A Review. NeuroRehabilitation.

[31] Yuhas P.T., Shorter P.D., McDaniel C.E., Earley M.J., Hartwick A.T.E. (2017). Blue and Red Light-Evoked Pupil Responses in Photophobic Subjects with TBI. Optom. Vis. Sci..

[32] Ciuffreda K.J., Joshi N.R., Truong J.Q. (2017). Understanding the effects of mild traumatic brain injury on the pupillary light reflex. Concussion.

[33] Lowenstein O., Loewenfeld I.E. (1950). Mutual role of sympathetic and parasympathetic in shaping of the pupillary reflex to light; pupillographic studies. Arch. Neurol. Psychiatry.

[34] Dukhayer S., Bushuieva N.M., Slobodianyk S.B. (2020). Pupil Response to Accommodation and Convergence in Healthy Children and Adolescents of Various Age Groups and Autonomic Nervous System Tone. J. Ophthalmol..

[35] Kaltsatou A., Kouidi E., Fotiou D., Deligiannis P. (2011). The use of pupillometry in the assessment of cardiac autonomic function in elite different type trained athletes. Eur. J. Appl. Physiol..

[36] McDougal D.H., Gamlin P.D. (2015). Autonomic control of the eye. Compr. Physiol..

[37] Anderson H.A., Parks S.M., Kulp M.T., Mitchell G.L. (2025). Classification of accommodative insufficiency by monocular subjective push-up test is poorly predictive of monocular objective amplitudes in children and young adults. Ophthalmic Physiol. Opt..

